# The Deliverances of the Retina*Read before the Alumni of the Buffalo Medical College, Feb. 21st, 1882, by F. W. Abbott, M. D.

**Published:** 1882-04

**Authors:** F. W. Abbott


BUFFALO

### Medical and Surgical Journal.

VOL. XXI. APRIL, 1882. No. 9.

### Original Communications.

### THE DELIVERANCES OF THE RETINA.*

*Mr. President and Gentlemen of the Alumni Association:*

When we examine the structure and functions of the visual apparatus, we find that the rays of light pass through the transparent refracting media of the eye,—the cornea, aqueous humor, lens and vitreous,—and impinge upon the retina. We also find that the optic nerve, which in structure cannot be distinguished from other nerves, connects the brain with the retina. The natural inference is that in the retina rays of light are transformed into something which can be carried along by ordinary nerve fibres, *i. e.* the retina receives rays of light and delivers over to the optic nerve fibres something which answers to what we call irritation of nerves. Now, these retinal processes, the transformation of light rays into the irritations which are delivered over to the optic nerve fibres by the retina, which I have entitled for brevity "The Deliverances of the Retina," form the subject of my paper. The time at my disposal is so short that I cannot stop to prove many points which may seem to some of you to demand proof, but you may rely upon my assertions of

* Read before the Alumni of the Buffalo Medical College, Feb. 21st, 1882, by F. W. ABBOTT, M.D.

fact; as to the theories which I advance, you are to be the judges whether the facts sustain them.

A glance at the illustrations will give us the necessary anatomical facts for our purpose. Fig. I represents a section of the retina; 1 the internal limiting membrane which lies upon the vitreous humor; 2, the layer of optic nerve fibres; these radiate in all directions from the entrance of the optic nerve; 3, the layer of ganglion cells; these are in every respect like the ordinary multipolar cells of the rest of the cerebro-spinal system; each of these cells is connected by one process with a fibre of the nerve-fibre layer; the other processes, according to the most careful observers, are lost in the next layer; 4, the inner granular layer. The other layers: 5, the inner corpuscular; 6, outer granular; 7, outer corpuscular; and 8, external limiting, need not detain us. No. 9, the layer of rods and cones, and No. 10, the pigment layer, demand from us especial attention, as the principal point of this paper turns upon the structure and relations of these two layers. This ninth layer is composed entirely of these rods and cones, which stand closely packed together like a mosaic. The cones are shaped very much like a champagne bottle. In the fovea centralis, which lies directly opposite the centre of the cornea, where vision is most distinct, cones alone are found. As we recede from this point toward the ora serata, the rods become more numerous. At first every cone is surrounded by a single layer of rods, then by a double layer, &c. The structure of the cones and rods seems to be identical. Each is composed of an inner body, slightly striated, and an outer body, which is perfectly transparent. In the cones the inner body is considerably larger than the outer body, while the rods preserve the same diameter throughout their whole length. At the fovea, as said before, cones alone are found, and here the transparent outer body is very much elongated.

The pigment layer has until recently been considered rather as a layer of the choroid than of the retina; but the latest authorities, as Schultze and Schwalbe, consider it a true retinal layer, and, as we shall see farther on, it is a very important one. This pigment layer is composed of flat pigment cells which send processes down between the cones and rods, and these adhere so closely to the pigment that their outer bodies often remain sticking in the pigment when we separate these two layers. This is the particular anatomical point to which I wish to call your attention. At the fovea the entire interspace between the transparent outer bodies of the cones is filled in with this dark pigment. It is as though you stood a quantity of champagne bottles as close together as possible and sifted sand over them. Comparatively little could lodge between the bodies of the bottles, but the large interspaces b tween the necks would hold more, until, as the surface of the sand approached the corks, there would be more sand than glass. So at the fovea each long, transparent, outer body of a cone is imbedded in a mass of pigment which fits it as a glove fits a finger, and separates it from its neighbors on all sides by a dark, opaque barrier. Beyond the tips of the cones here, and of the rods and cones of the rest of the retina, the pigment cells form a dark continuous layer which lies against the choroid.

The layer of rods and cones is the percipient layer of the retina. The rays of light pass through the other layers without making any impression. It is only when they reach this place that they excite sensations. Hence the rods and cones are evidently the terminal organs of the optic nerve. But in spite of the most careful and patient microscopic examinations, a direct connection between a cone and an optic nerve fibre has never been discovered. Fig. II shows us what our æsthetic friends would call a conventionalized representation of the nervous connections so far as they have been seen. Upon the inner side of the retina, as we saw, the nerve fibres connect directly with the ganglion cells, which send processes to the external granular layers, where they are lost and can be traced no further. Each rod and cone connects with a separate molecule of the external molecular layer, and from this molecule a filament extends to the external granular layer, where it disappears. So we see that direct nervous connection is lost between the external and internal granular layers.

So much for the anatomy of this extremely delicate membrane, whose ten layers added together are less than $\frac{1}{100}$ of an inch thick.

The study of the physiology of the retina has to be entirely subjective. Microscope and test tube cannot tell us what it does. For a knowledge of its workings we have to depend entirely upon perceptions and sensations. We know that we see with our eyes. We know also that if we want to see anything distinctly, we must look directly at it; but we also take in a large field of vision, so that while we see with distinctness the object at which we are directly looking, we see with less clearness the objects which lie about it. Studies of the refracting media of the eye have taught us that for every object in the field of vision there is a corresponding picture thrown onto the retina, like the picture which is formed on the screen in the camera. A comparatively simple calculation tells us the relation between the size of the observed object and the size of its retinal picture, so that, if we measure the smallest objects which we can see, or distinguish, we can easily calculate the size of the retinal pictures which these minute objects make. Of course we cannot measure the size of observed objects in feet and inches, for a near object occupies relatively a larger space in the visual field than a distant one. So for these observations we note how many degrees in the arc of a circle our objects cover, which circle has the eye for its centre; *e.g.* a wafer about one-fifth of an inch in diameter held a foot away from the eye covers an arc of one degree in the circle, whose radius is one foot. You see at once that this wafer, one-fifth of an inch in diameter, at one foot covers as large an area in the visual field as an object whose diameter is twenty times as great at twenty feet from the eye, which would also cover an arc of one degree in a circle whose radius is twenty feet, and the wafer and a medal four inches in diameter at these distances throw pictures of the same size on the retina. Now the sharpness of vision is measured by the distance which must intervene between two objects in the visual field, in order that they may be distinguished, and this distance has been accurately measured by different observers. Hooke found that he could no longer distinguish two stars when their distance apart was less than 60″. E. H. Weber found in his examinations that two white lines had to be 73″ apart to be distinguished. Helmholz could see two lines as separate when the angle between them was 64″. Confusion has arisen in the minds of some in considering this subject, by not observing the distinction between to *see* and to *distinguish.* We can see a fixed star, a mere point of light, of which the most powerful telescope can make nothing but a mere point without length or breadth. But if two stars approach each other so that the distance between them is less than 60″, they can no longer be distinguished as two stars, but appear to be one single bright spot. We can see with ease an electric light five miles away, where it would cover only a fraction of a second of the visual field; but at this distance two electric lights would need to be about four feet apart to enable us to distinguish that there were two lights instead of one single source of illumination.

As we have just said, we can calculate the size of the retinal pictures of observed objects, and so we can find out the distance which separates the retinal pictures of two objects which can barely be distinguished. Now, if we compare the distance upon the retina between the retinal pictures of two barely distinguishable objects with the thickness of the cones at the fovea, we shall find that these two pictures must be far enough apart to fall on two separate cones; and also that if two retinal pictures are so close together that they fall on one cone, we no longer get two sensations, we are no longer conscious of seeing two objects, the two pictures which fall upon one cone are no longer distinguished as two by us, but are merged together and produce one sensation. We explain this on the theory that each cone has one nerve fibre which connects it with the brain, so that one impression, or several simultaneous impressions on the same cone, can irritate only one nerve fibre and therefore can produce but one sensation.

As retinal pictures recede from the yellow spot and approach the ora serrata, they need to be farther apart to produce two sensations. In other words, two objects, as they approach the boundaries of the field of vision, need to be farther apart to be distinguished than they do when they are in the direct line of vision. This statement you can easily verify by looking at the points of your dressing forceps held half an inch apart at arm's length. When you look directly at them, you distinguish two points. If you keep the eye still and move the forceps to one side, you can still see the forceps, but soon cannot distinguish the two points or tell whether the forceps are partly open or shut together.

The Germans call the extent of surface which gives only one sensation, whether the impressions upon it be one or many, an *Empfindungskreis;* we will translate this happy term by sensation area, and defining it again, we say a sensation area is that extent of a sensitive surface which gives but one sensation, whether one or many simultaneous impressions are made upon it. The skin has sensation areas which vary in size. I touch the tip of my finger with the points of these dividers one-fourth of an inch apart, and feel the two points. I have two sensations, have touched two sensation areas. I touch the back of my hand with the same two points. I no longer distinguish two points, but feel only one touch—I have only one sensation. Evidently the sensation areas are larger here than at the pulp of my fingers. I put two white beads on the points of my dividers and look directly at them. I see two beads. I get two sensations. Evidently the pictures of these two beads have fallen on two separate sensation areas of my retina, and what we have already gone over teaches us that these pictures must have fallen on two separate cones. As I move the dividers away from the line of direct vision, I soon find that I can no longer distinguish two beads, but can easily see a white object. The pictures of these beads have receded from the fovea; they have evidently both fallen on the same sensation area, and the two pictures afford me only one sensation.

So much for facts, which are stupid things, because they can excite no discussion. The theories which follow may admit of considerable argument.

We have seen that at the fovea the sensation areas correspond with the size of the cones and grow larger as we leave this point. We have seen that the cones are less frequent as we leave the fovea, being surrounded at first by a single circle of rods, then by a double circle, &c. The layer of ganglion cells is thickest at the yellow spot where they lie six or eight deep. They become more scarce toward the ora serrata, where they are quite isolated from each other. Optic nerve fibres have been traced to the ganglion cells, and processes from them pass into the granular layer, but can be traced no further. Upon these facts is built the theory that each cone with its surrounding rods forms one sensation area, which connects with one optic nerve fibre through one single ganglion cell. So that pencils of light rays which fall upon a cone or any of its surrounding rods, send a sensation along the same nerve filament to the brain. If several rays fall upon a cone and its surrounding rods at the same time, only one sensation is aroused, but this is the resultant of the different impressions upon this one area.

This question may be asked: If the number and location of sensation areas correspond to the number and position of the cones, why consider the rods, which are not shaped like the cones, as having the same functions? The answer is that careful observation has shown that the peripheral field of vision does not show such hiatuses in viewing minute objects like the stars, as it would show were the cones alone sensitive to light.

We can now begin to see our way toward a definition of our subject, and say that the deliverances of the retina are a distinct irritation of a separate optic nerve fibre for every sensation area upon which a ray of light falls. The irritations of these different nerve fibres are distinguished at the seat of consciousness and produce there distinguishable sensations.

These retinal sensation areas are sensitive to or excited by light. Light consists of undulations or waves of different lengths, and these waves of different lengths affect these sensation areas differently, so that for the different wave lengths they deliver over different irritations to the nerve fibres. These different irritations we distinguish in consciousness and call them different colors. When light rays, whose waves are 0.0006878 m m long, and of which there are 450 billions in a second, strike the retina, we say that we see a red color; if they are only 0.0003928 m m long and 790 billion strike the eye in a second, we see a violet color, and all the colors of the spectrum are composed of rays whose wave lengths are between these two extremes. Since light waves which are more than 0.0006878 m m in length, do not irritate the retina, and those which are shorter than 0.0003928 m m, also do not irritate the retina, we see that all the different sensations which we receive through the medium of the retina are caused by light waves which vary in length by less than 0.0006878 m m—0.0003928 m m=0.0002950 m m. So we learn, further, that the deliverances of the retina are irritations of the optic nerve fibres, whose differences in quality are caused by differences of less than 0.0002954 m m in the length of the waves which impinge on the retina.

But the most interesting part of our subject, and the part for which this paper was written, remains to be considered; and that is, whereabouts in the retina are light rays changed to nerve irritations. The opinion is generally held that the cones and rods are directly sensitive to light rays, and that these light waves, in passing through the cones, directly irritate them. This opinion I hold to be an error, and would present another theory which seems to me to explain the facts much more satisfactorily.

This theory is that the rays of light do not irritate the rods and cones directly, as they pass through them, but that these light rays, after passing through the transparent layers of the retina, without doing any work, set up a molecular motion in the pigment layer, and this molecular motion of the pigment beating against the cones and rods irritates them.

To this theory I would invite your careful attention, for if it is true, it will work radical changes in our ideas of the Physiology of vision. The first argument for the support of this theory is, that the cones which have been said to be irritated by light rays are perfectly transparent, *i. e.*, they allow at least ninety-nine per cent. of the light to pass through them unchanged. According to the well-known laws of the correlation and conservation of forces, whenever a force does any work, it diminishes or disappears. When a falling stone reaches the ground, heat is developed, but the motion ceases. The pumps at the water works lift 20,000,000 gallons of water a day, but the boilers do not give off as much heat as they would with the combustion of the same amount of coal if they did no work. Some of the heat has been transformed into motion. When the sun's rays fall upon a transparent window pane, some are reflected, most of them pass through and the pane is not warmed, it remains unaffected. If the rays then fall upon a carpet, a few are reflected, and we see a bright spot, the rest are absorbed, and the spot is warmed. So we see that whenever the sun's rays do any work, they disappear as sun's rays, and that an object which is transparent to them, is unaffected by them. It is unphilosophical to suppose that the retina is the sole exception to this universal law, and that here the particular part which is to be affected by the rays of light, should be the most transparent to those rays and allow them to pass through practically undiminished.

But it may be objected, allowing that ninety-nine per cent. of the light does pass through the cones, is not the one per cent. sufficient to irritate the cone and give us our sensation? Of course it is impossible to say "no" with mathematical certainty, for the equivalents of light and nerve irritation have never been calculated; but since faint starlight suffices to excite the retina, while sunlight or the dazzling glare of electricity is necessary to print a picture on the photographer's plate, analogy and reason tell us that it is impossible, using Kühne's expressive phrase, that ninety-nine per cent. of the faint starbeams have gone to death or been uselessly absorbed in the pigment, and that one per cent. only has been utilized in doing the work for which the retina was intended.

In the next place, if this theory is true, we are not obliged to attribute special properties to the optic nerve fibres and the rods and cones, which isolate them from the rest of the nervous system.

Light rays irritate no other nerves. Other nerves are irritated by being touched. The terminal filaments of the auditory nerve are irritated by the molecular motions which the sound waves set up in the fluid of the cochlea in which these terminal filaments float. I am not sure that I quite understand Miller's theory of the "specific energy" of the nerves of special sense; but this theory seems to me to do away with the necessity of supposing a specific energy for the terminal filaments of the optic nerve, for such we must consider that the rods and cones really are, which specific energy makes them alone sensitive to light rays. This theory enables us to consider the optic nerve as identical with other nerves in function as it is in structure, and sensitive to the same irritations. Other nerves are sensitive throughout their whole length. A blow upon the ulnar nerve at the elbow hurts like an injury to the little finger. The old theory would have us believe that the light rays pass through the fibres of the optic nerve which form the inner layers of the retina, without irritating them, and only irritate the termini at the outer layer. Our theory says light rays as such do not affect nerve fibres at all, and can be transformed into motion only in an opaque substance like the pigment where they disappear as light rays.

But, some one says, the most powerful beams of light, if unaccompanied with heat, never excite sufficient molecular motion in the skin to arouse a sensation, how can this molecular motion arouse a sensation in the retina. I would say the anatomical relations of nerve and pigment and the direction from which the rays come are so different in skin and retina that no comparison is possible. In the eye the lens sorts out the light rays which fall upon it and directs those which come from one point of the visual field, and hence are of one color to one point of the retina, here they pass undiminshed through various strata and fall directly upon the dark pigment which lies upon the cones as your glove hugs your fingers, and if they are changed to motion this motion beats directly upon the cones. We might as well argue a specific energy for the fingers, because we can feel the kid of our gloves, while cannot tell by the sensations of our shoulder blades what our overcoat is lined with, as to attempt to argue from an ordinary sensitive nerve covered from light by derma and cuticle to the closely wedded cone and pigment. Many other arguments might be adduced, but my time is up. Of course this theory is not original. I got the idea from Prof. Fick's article on the eye in the *Handbuch der Physiologie*, edited by Prof. Hermann, of Zurich. I have only worked out some of the arguments in its favor. This theory has not, to my knowledge, been yet advanced in any English speaking authority.*

I love my Alma Mater and am jealous for her fame. I present this imperfect sketch that her alumni, whose studies have not led them into paths of special research, may yet know a little of what is going on among those who are cultivating these special fields.

---

*Since this paper was read, my attention has been directed to Draper's Physiology, in which it is stated that light rays cannot make an impression on the transparent parts of the retina, but that passing through these they are changed to heat in the pigment, and that the variations of temperature in the pigment affect the contiguous nervous elements so as to cause sensations of various colors. Heat is *a* mode of motion, but not the only one, and I do not think that Dr. Draper proves that it is *the* mode of motion which is set up in the retinal pigment by the light rays. **F. W. A.**

